# Assessment of complications and short-term outcomes of percutaneous peritoneal dialysis catheter insertion by conventional or modified Seldinger technique

**DOI:** 10.1080/0886022X.2021.1925296

**Published:** 2021-06-07

**Authors:** Yun Zou, Yibo Ma, Wenying Chao, Hua Zhou, Yin Zong, Min Yang

**Affiliations:** a Department of Nephrology, The Third Affiliated Hospital of Soochow University, Changzhou, Jiangsu Province, China; b Department of Ultrasound, The Third Affiliated Hospital of Soochow University, Changzhou, Jiangsu Province, China

**Keywords:** Peritoneal dialysis, percutaneous catheterization, Seldinger technique, ultrasound guidance

## Abstract

**Objective:**

To explore the efficacy and short-term complications of a modified technique to percutaneously insert a peritoneal dialysis catheter.

**Methods:**

We reviewed the outcomes of 94 patients who underwent peritoneal dialysis catheterization between October 2017 and April 2020. Of these, 47 cases were placed by a conventional Seldinger technique, whereas 47 cases were placed by a modified technique based on the Seldinger method. The success rates of the catheter insertion and three-month postoperative complications were compared between these two groups.

**Results:**

The catheter insertion success rates were comparable between the two groups: 93.6% in the conventional technique group and 97.9% in the modified technique group (*p* = 0.307). The incidence of postoperative catheter migration was lower using the modified technique (4.3%) than the conventional technique (18.3%) (*p* = 0.037). None of the patients in the modified technique group had postoperative dialysate leakage, whereas this occurred in 9.0% of patients in the conventional technique group (*p* = 0.036). There were no statistically significant differences in the incidence of postoperative bleeding, infection, or visceral damage between the two groups.

**Conclusions:**

The modified Seldinger technique for percutaneous peritoneal dialysis catheter insertion reduced the short-term postoperative complications of catheter migration and dialysate leakage, with a comparable successful catheter insertion rate compared with the conventional Seldinger technique.

## Introduction

Peritoneal dialysis (PD) is one of the renal replacement therapies for patients with end-stage renal disease. It has the advantages of home-based treatment, continuous toxin removal, persistent protection of residual renal function, and low treatment costs. Successful placement and free fluid flow through the PD catheter are essential for the satisfactory performance of PD. The primary reasons for technical failure and patient withdrawal from PD have been displacement and obstruction of the PD catheter, typically mechanical complications following catheter placement, which affect the efficacy and safety of this dialysis technique [1,2]. A PD catheter is placed utilizing one of several approaches: open surgical, laparoscopic, or percutaneous. The percutaneous approach has the advantages of a short procedure, small incision, and relatively straightforward technical requirements which have resulted in an increasing number of hospitals embracing this technique. However, similar to all other procedures, percutaneous PD insertion also carries certain risks, such as bleeding, visceral injury, and dialysate leakage [3]. In this study, we modified the traditional Seldinger puncture method by (1) replacing the puncture needle with a pneumoperitoneum needle, (2) employing preoperative and intraoperative ultrasound examinations to assess and guide catheter placement, (3) a short incision of the anterior rectus sheath for catheter insertion, (4) water-tight suture closure of the rectus sheath, and (5) a hard guidewire in the catheter to guide final placement. The intent of the present study was to investigate the success of catheter placement and short-term complications comparing the modified technique to the traditional Seldinger technique.

## Materials and methods

### Study population

A retrospective study was conducted on 94 patients who underwent percutaneous peritoneal dialysis catheterization by either a standard or modified Seldinger method in the First People's Hospital of Changzhou City from October 2017 to February 2020. From October 2017 to January 2019, 47 patients underwent conventional Seldinger percutaneous peritoneal dialysis catheterization. Due to a high number of complications, we modified the procedure starting in February 2019, and 47 patients underwent the modified procedure through February 2020. The incidence of complications in the two groups within three months post-procedure was compared. The exclusion criteria were as follows: patients ≤16 years old and patients who underwent laparoscopic or surgical catheter placement. MedComp peritoneal dialysis tube puncture kits (Medical Components, Harleysville, PA, USA) were used in all patients using a straight double-cuff Tenckhoff catheter. Two experienced nephrologists and one sonographer were responsible for all percutaneous peritoneal dialysis catheterizations throughout the study period. The study was approved by the Ethics Committee of the Third Affiliated Hospital of Soochow University, China (registration number: #08/2020).

#### Catheter insertion by conventional technique

The insertion site was selected at a point 10 cm above the pubic symphysis and 1–2 cm next to the anterior midline of the abdomen. Preoperative ultrasound was used to avoid the underlying abdominal wall arterial blood vessels, as well as to measure the thickness of the rectus abdominis in order to estimate the length required for the insertion needle. Antibiotic prophylaxis using cefazolin 1 g I.V was infused 0.5–1 h pre-procedure, and the bladder was emptied. The patient was positioned supine and the needle insertion site was locally anesthetized with 1% lidocaine. A 2–3 cm incision was made, the subcutaneous tissues blunted dissected to reach the anterior sheath of the rectus abdominis. A sheathed puncture needle was inserted into the peritoneal cavity through the rectus abdominis at a 45-degree downward angle. Following infusion of 500 mL of saline, the guidewire was passed through the puncture needle into the pelvis. A dilator and a peel-away sheath were next introduced over the guidewire to reach the middle of the pelvic cavity. The guidewire and dilator were removed and the dialysis catheter was inserted through the sheath into the peritoneal cavity. The peel-away sheath was removed and the inner cuff was forced through the rectus abdominis sheath. Free drainage of saline confirmed the correct position of the dialysis catheter. The catheter was guided downward by a tunneling tool through the subcutaneous tissue, in an arc from top to bottom, with the skin exit site 6–8 cm from the puncture point. The tunneling tool was detached from the end of the Tenckhoff catheter and a titanium adapter and transfer set were applied. After confirming that there was no seepage of blood or fluid, the subcutaneous tissue and skin were sutured in layers.

#### Catheter insertion by modified technique

Five improvements were made to the conventional Seldinger method: (1) preoperative ultrasound to locate the abdominal wall arteries and intraoperative ultrasound guidance throughout the procedure; (2) a pneumoperitoneum needle replaced the puncture needle in the PD kit for skin puncture; (3) a 1–1.5 cm longitudinal incision was made in the anterior sheath of the rectus abdominis to expose the muscle, ensuring atraumatic separation of the muscle fibers for placement of the cuff; (4) water-tight placement of 2–3 stitches (instead of purse-string suture) to close the anterior sheath around the catheter; and (5) use of a hard guidewire in the dialysis catheter when introduced into the peritoneal cavity through the peel-away sheath (similar to an open surgical technique).

#### Postoperative plan

PD was initiated immediately on all patients after PD catheter insertion with a Baxter Peritoneal Dialysis solution (Lactate-G1.5%).All patients were treated with intermittent peritoneal dialysis (IPD) with a basic ESKD PD prescription of four exchanges and 4 h dwell time. The amount of remaining abdominal fluid was gradually increased from small doses of 800 mL for 1–2 days following PD insertion to 1000 mL for 3–4 days, 1500 mL for 5–7 days, and on day 8, 2000 mL of peritoneal dialysate was infused each time and standard continuous ambulatory peritoneal dialysis (CAPD) was started. All PD catheter insertions were performed for hospitalized patients following which the patients were discharged after 5–7 days of observation.

### Outcome measurements

Enrolled patients were grouped into either the conventional technique group or the modified technique group. Successful placement of the dialysis catheter was based on free intraoperative drainage of the fluid through the PD catheter, and placement of the tip of the dialysis catheter in either the pouch of Douglas or the right or left iliac fossa confirmed by abdominal x-ray performed the day after the catheter insertion. If the use of a percutaneous needle technique failed, the patient was switched to open surgery, because in our center, both percutaneous puncture and open surgery were performed under local anesthesia and independently by nephrologists, while laparoscopic surgery required doctors with endoscopic skills to perform under general anesthesia. Therefore, it was easier to switch to open surgical therapy for patients who were failed percutaneous implantation without the need to change the anesthesia plan and the team.

Complications were recorded within three months after the procedure including bleeding, catheter migration, omental wrapping, dialysate leakage, visceral damage, hernia, wound infection, peritonitis and tunnel infection. Catheter migration was defined as displacement of the catheter tip from the pelvis into the abdomen above the posterior border (sacral promontory) of the pelvic brim on abdominal pelvic radiograph. Routine abdominal-pelvic radiographic examinations were performed the day after catheter insertion and three months post-procedure, or immediately whenever outflow volume decreased.

### Statistical analysis

All statistical analyses were conducted with Statistical Package for the Social Sciences (SPSS) software program, version 25.0 (SPSS, IBM, Armonk, NY, USA). All data were checked for normality of distribution using the Kolmogorov–Smirnov test of normality. Normally distributed data were represented as the mean ± standard deviation. Non-normally distributed data were represented as the median (inter-quartile range [IQR]). The Mann–Whitney test was used for non-normally distributed continuous variables. Student’s t test was used to test for differences between groups for normally distributed continuous variables. The chi-square test was used to evaluate differences in incidence. A value of *p* < 0.05 was considered statistically significant.

## Results

### Baseline characteristics of study participants

The study comprised 94 patients who underwent PD catheter insertion including 56 male and 38 female patients, age 45.3 ± 15.4 years old (Table 1). The causes of the ESKD were chronic glomerulonephritis in 61, 19 with diabetic nephropathy, 8 with hypertensive nephropathy, 2 with ANCA-associated vasculitis, 3 with lupus nephritis, and 1 with renal amyloidosis. The number of patients in the conventional technique group and the modified technique group was equal with 47 cases each. There were no significant differences between the two groups in gender, age, body mass index (BMI), history of diabetes, history of abdominal surgery, thickness of the rectus abdominis and laboratory test results.

**Table 1.** Demographic and biochemical characteristics of the 94 patients undergoing peritoneal dialysis catheterization.

Characteristics	All patients (*n* = 94)	Conventional group (*n* = 47)	Modified group (*n* = 47)	*p* Value
Age, year	45.3 ± 15.4	46.0 ± 17.2	44.5 ± 13.6	0.642^a^
Male gender, *n*%	56 (59.6%)	26 (55.3%)	30 (63.8%)	0.401^b^
Diabetes, *n*%	19 (20.2%)	9 (19.1%)	10 (21.3%)	0.797^b^
BMI, kg/m^2^	21.3 (19.4, 21.3)	21.2 (19.8, 25.8)	21.8 (19.4, 24.2)	0.484^c^
History of abdomen surgery, *n*%	15 (16.0%)	5 (10.6%)	10 (21.2%)	0.159^b^
Hemoglobin, g/L	81.1 ± 15.0	80.5 ± 13.1	81.7 ± 16.9	0.697^a^
Creatinine, umol/L	780.5 (660.0, 983.0)	784.0 (643.0, 963.0)	777.0 (667.0, 990.0)	0.553^c^
BUN, mmol/L	30.2 ± 10.6	29.5 ± 12.1	31.1 ± 8.9	0.465^a^
Albumin, g/L	34.4 ± 5.9	33.9 ± 6.3	34.9 ± 5.4	0.433^a^

BMI: body mass index; BUN: blood urea nitrogen.

^a^ Student’s *t* test.

^b^ Chi-square test.

^c^ Mann–Whitney test.

### Comparison of successful dialysis catheter insertion between the two groups

In the conventional technique group, 44 patients successfully completed the dialysis catheter insertion with a success rate of 93.6%, whereas in the modified technique group, 46 patients successfully completed the catheter insertion, 97.9%, not significantly different (*p* = 0.307).

Of the three patients with unsuccessful insertion in the conventional technique group, two patients failed with repeated attempts due to obstructed guidewire insertion and were converted to the open surgical technique. The third patient had guidewire obstruction despite repeated dialysis catheter insertion attempts. Notably, a mesenteric artery had been injured and was bleeding when the patient was managed with open surgery.

For the one patient with unsuccessful insertion in the modified technique group, ultrasound examination revealed thickening of the extraperitoneal tissue with saline infusion that suggested the dialysis catheter was placed in the extraperitoneal space. Following several failed attempts of percutaneous catheter insertion, the patient underwent open surgical catheter placement.

### Comparisons of the postoperative complications between two groups

In the conventional group, 8 patients experienced catheter migration confirmed radiographically within three months of catherization. Of the 8, 7 were self-reset with conservative treatment, such as promoting intestinal emptying and climbing up and down stairs, and 1 patient improved after reinserting the catheter laparoscopically. In the modified group, 2 patients experienced catheter migration; one was self-reset after conservative treatment and the other improved after laparoscopic surgery. Both catheter migration and omental wrap were found during the laparoscopic procedure. The difference between the two groups was statistically significant (Table 2).

**Table 2.** Comparisons of postoperative complications between the conventional and modified technique groups in 90 patients with successful percutaneous peritoneal dialysis catheter insertion.

Complications	Conventional group (*N* = 44) [*n* (%)]	Modified group (*N* = 46) [*n* (%)]	*p* Value
Bleeding	0 (0.0)	1 (2.2)	0.325^a^
Catheter migration	8 (18.2)	2 (4.3)	0.037^a^^,^*
omental wrapping	4 (9.0)	3 (6.5)	0.649^a^
Dialysate leakage	4 (9.0)	0 (0.0)	0.036^a,^*
Peritonitis	3 (6.8)	3 (6.5)	0.955^a^
Fistula infection	2 (4.5)	0 (0.0)	0.144^a^
Visceral injury	0 (0.0)	0 (0.0)	
Hernia	0 (0.0)	0 (0.0)	
Wound infection	0 (0.0)	0 (0.0)	

*p < 0.05.

^a^ chi-square test.

In the conventional technique group, 4 patients (9.0%) had peritoneal dialysate leakage after the procedure while no patients in the modified group had postoperative leakage. The difference between two groups was statistically significant (Table 2).

There were 4 cases in the conventional group and 3 cases in the modified group (including 1 case with catheter migration) that required laparoscopic correction of omentum wrap (not statistically significant). In the modified technique group, a single patient experienced tunnel bleeding, which was controlled with local compression and hemostatic medications. There were no statistically significant differences between the two groups in complications such as bleeding, abdominal visceral damage, infection, and hernia (Table 2).

## Discussion

In our city, most patients with ESKD are unwilling to undergo an early operation to create an arteriovenous fistula or insert a PD catheter due to economic and cultural reasons. In our PD center, greater than 95% of ESKD patients started PD therapy urgently and initiated PD immediately after the PD catheter was inserted. A cohort study of 922 Chinese patients showed that urgent-start PD was a safe and practicable approach for eligible patients with uremia, with an acceptable frequency of mechanical complications [4]. The research from ÁkosPethő also showed that percutaneous peritoneal dialysis catheter insertion that minimized the skin incision, was an effective and safe way to start PD immediately without having to delay for the requisite wound healing of 6–8 weeks [5].

The successful placement of a PD catheter is essential for PD performance [6]. Catheter-related complications may lead to technical failure and reduce the long-term effectiveness of the catheter. Approximately 20% of patients initially started on PD had to switch to hemodialysis due to catheter-related complications [7], which commonly included infectious complications such as peritonitis and tunnel infection, as well as mechanical complications such as catheter migration, omental wrapping, leakage, bleeding, and hernia [8]. Mechanical complications were usually related to failure of the actual PD catheter placement which compromised both the catheter’s lifespan and patient survival [8–10].

**Figure 1.**
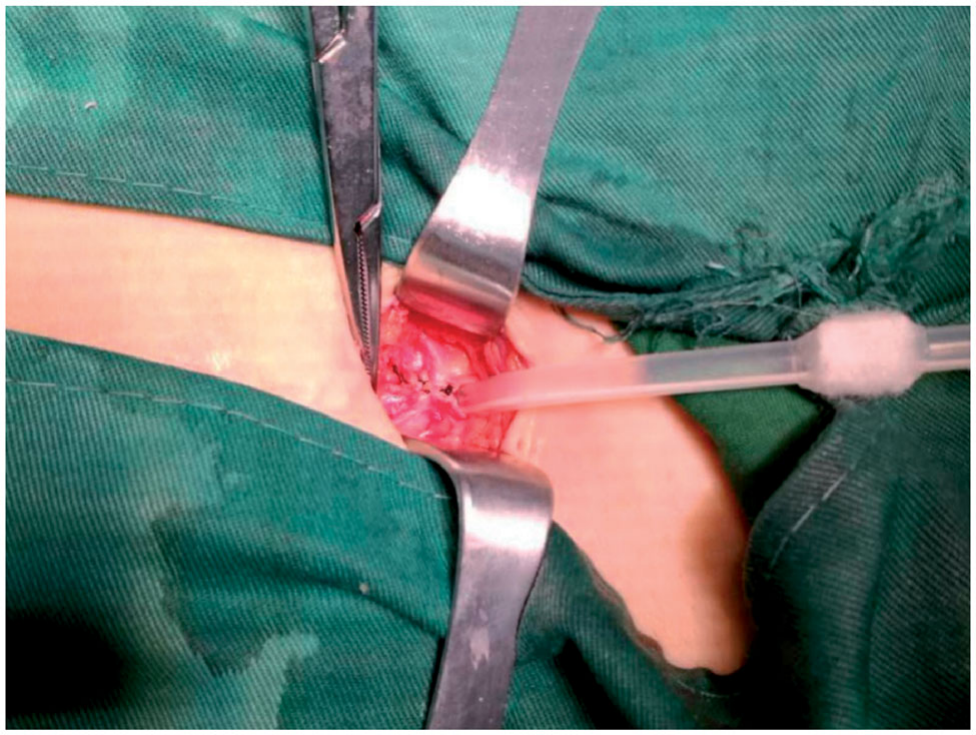
Put 2-3 stitches from the tail to the cephalic direction to suture the anterior sheath of rectus abdominis.

Both catheter migration and blockage are collectively referred to as catheter dysfunction, which is the most common mechanical complication during PD. Almost 90% of catheter dysfunction has occurred in the first 12 weeks following the procedure (50% occurred in the first four weeks) [11,12]. The intraperitoneal aspect of conventional percutaneous catheter insertion is performed blindly which makes it impossible to visually direct the catheter appropriately into the pelvic cavity. Therefore, patients might experience discomfort in the lower abdomen if the catheter is placed too deeply and stimulates the rectum. Conversely, if the catheter placement is too shallow, the tip may become fixed within the omentum causing drainage failure. In our study, due to relatively high frequency of catheter migration in the conventional group, we used ultrasound as our ‘perspective eye’ in the modified technique group, which could not only guide us to effectively avoid blood vessels during the operation, but also observe whether the tip of the peritoneal dialysis catheter was placed in the Douglas fossa (Figure 1). Additionally, a hard guidewire could still be used to adjust the position of the catheter to make sure that its tip reached the optimal drainage position, even after removal of the peel-away sheath. The result, catheter migration was successfully reduced.

After the PD catheter is placed in the abdominal cavity, due to the propensity of the omentum to adhere to foreign bodies, the catheter can be surrounded and obstructed by the omentum, an important cause of catheter dysfunction [13,14]. In our study, 7 patients in the conventional group and 1 patient in the modified group who were able to correct catheter migration with conservative measures, probably related to simple catheter tip migration rather than omentum wrapping. In laparoscopic and open surgery, either resection or folding and fixation of the long greater omentum is a common method to prevent omentum wrapping. These two methods are not available when using percutaneous PD catheterization. Methods to reduce omentum wrapping in percutaneous placement and whether a delay in catheter use for 10−14 days after placement can reduce the incidence of omental wrapping may be worthwhile exploring in future studies.

In peritoneal dialysis catheterization, straight and coiled-tip catheters are the most commonly used catheters, but the best choice is controversial. Recent studies have shown that use of a straight Tenckhoff catheter has a significantly lower rate of catheter dysfunction or drainage failure than with coiled catheters [15–17]. However, these studies were all based on the technique of open surgery; further study seems indicated in percutaneous catheterization.

**Figure 2.**
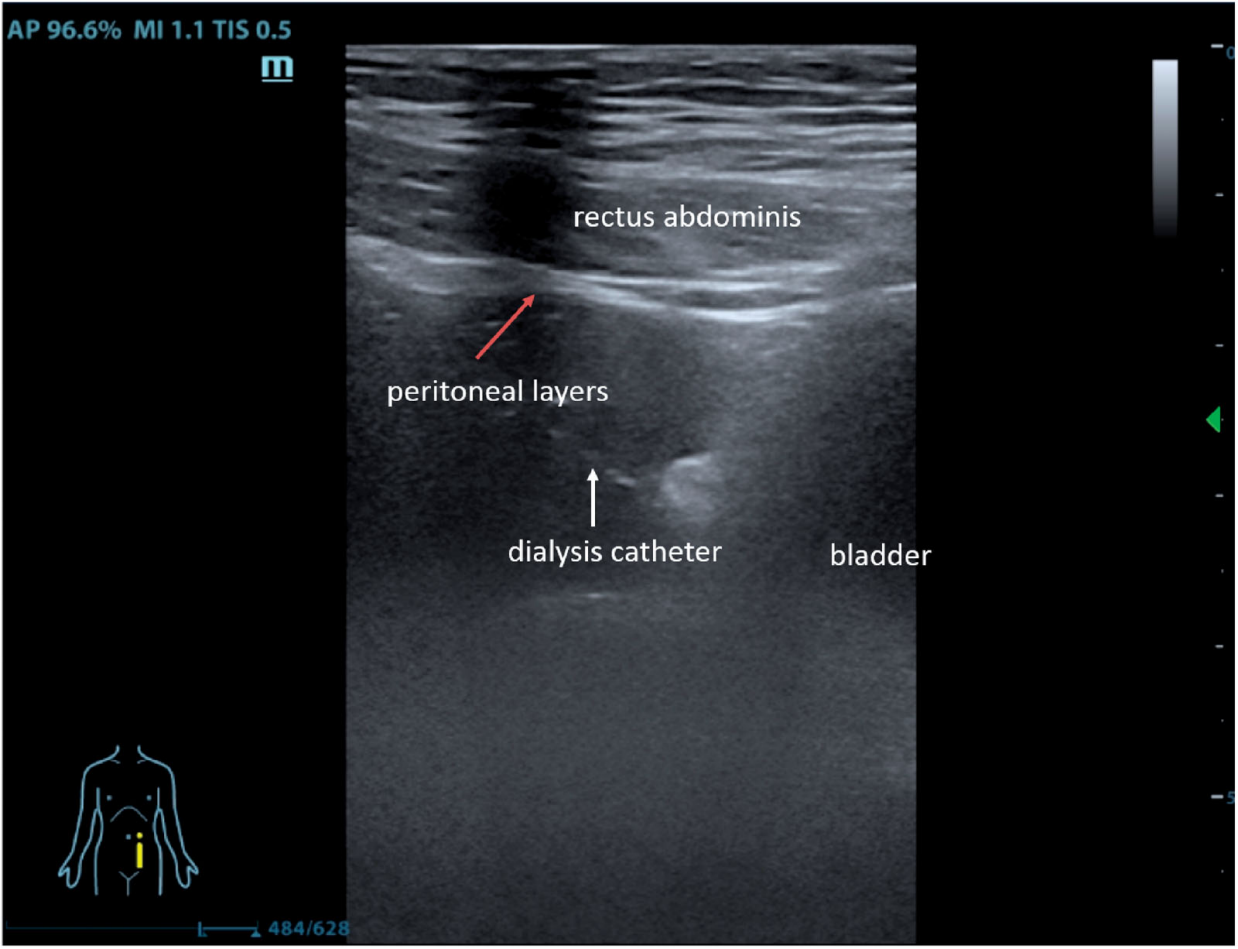
Ultrasound showed that the dialysis catheter was moved forward toward Douglas' fossa via a sheath (white arrow).

Peritoneal dialysate leakage is also a common early complication after percutaneous catheter insertion. The reported incidence ranges from 2.2 to 22% after percutaneous insertion, higher than the incidence following open surgical catheter placement [18,19]. This is attributed to such factors as internal cuff fixation failure in the muscular layer and poor closure from the purse-string suture. In our study, dialysate leakage occurred in 16.7% of patients in the conventional technique group which we thought was due to lack of rectus abdominis anterior sheath incision, failed fixation of the internal cuff into the muscles, and poor suturing quality. In the modified technique group, taking a lesson from the open surgical technique, a short incision in the anterior rectus sheath was made to expose the underlying muscle which enabled the internal cuff to be fully embedded within it. After positioning the catheter, the rectus abdominis sheath was sutured with 2–3 stitches progressing from inferior to superior, instead of using a purse-string suture (Figure 2). This modified technique was intended to not only prevent leakage, but also maintain the PD catheter in a downward direction from the outside to the inside of the pelvic cavity to reduce the risk of catheter migration.

The Seldinger technique is a blind penetration method with inherent complications, such as bowel perforation and bleeding. Bowel perforation is a serious early complication after PD catheter insertion with an incidence of about 1% [3]. Adhesion of the intestine to the abdominal wall, especially with repeated needle punctures could significantly increase the risk of bowel perforation and bleeding. The risk of adhesions in patients with previous abdominal surgery is reportedly as high as 70–90%, especially in patients with multiple prior laparotomies [20–22]. About 5% of patients even without a previous history of abdominal surgery can also have adhesions [23]. If there is relatively free movement of the small bowel by transabdominal ultrasound, the probability of significant adhesions is low, thereby reducing the risk of intraoperative bowel injury [24,25].

Abdominal bleeding is also a complication of traditional percutaneous catheter placement. One of the patients in the conventional technique group suffered intra-abdominal hemorrhage due to repeated abdominal wall punctures that damaged mesenteric vessels. Urgent open surgery was undertaken and the bleeding vessels were ligated. The insertion needle supplied in the percutaneous PD insertion kit is sharp, and causes only subtle tactile feedback when penetrating the peritoneum. With blind penetration, it is often nearly impossible to positively determine whether the puncture needle has entered the peritoneal cavity. Repeated punctures increase the risk of bleeding. To reduce this risk, we replaced the kit puncture needles with pneumoperitoneum needles in the modified group. The pneumoperitoneum needle has a spring protection device with a round blunt tip of a needle core. When encountering resistance, the needle core is pushed back into the needle sheath, allowing the sharp sheath to penetrate the peritoneum. Immediately upon penetration of the peritoneum, the resistance is gone, the spring is released and the blunt needle core pops through the sheath, protecting against injury to intraabdominal structures [26,27]. When saline was infused through the needle sheath, a low echogenic area could be observed between the intestines by ultrasound that indicated successful, atraumatic needle penetration. The concomitant applications of the pneumoperitoneum needle and ultrasound guidance could avoid repeated punctures and reduce complications. For one patient in the modified technique group, entry into the peritoneal cavity by a pneumoperitoneum needle was unsuccessful, and the patient was switched to the open surgical approach. The BMI for this patient was 29.2 kg/m^2^. The perceived sense of breakthrough that the pneumoperitoneum needle was thought to penetrate the peritoneum, was probably a misinterpretation of the needle only traversing the extraperitoneal fat layer, not the peritoneum. Therefore, we recommend usage of the pneumoperitoneum needle in patients with relatively thin abdominal walls and a BMI of 28 kg/m^2^ or less.

In summary, increasing numbers of dialysis teams have applied the Seldinger method to place PD catheters. It is important to identify and avoid the complications associated with this method. Our study demonstrated that concomitant applications of the pneumoperitoneum needle and intraoperative ultrasound guidance, together with small but important improvements in technique, reduced the risk of mechanical complications during catheter insertion. Moreover, our modified technique is straightforward to adopt, with less trauma, a lower risk of infection and bleeding, and warrants further validation.

The limitations of our study included a single-center retrospective approach, a small number of participants and short postoperative follow-up. Future prospective studies with a larger sample size and longer follow-up are required to confirm this study.

## Conclusions

Concomitant applications of the pneumoperitoneum needle and intraoperative ultrasound guidance, together with improved techniques based on the Seldinger method, reduced the risk for short-term postoperative mechanical complications of catheter migration and dialysate leakage. It is a safe and feasible technique for patients who require urgent-start peritoneal dialysis.

## References

[1] Crabtree JH. Peritoneal dialysis catheter implantation: avoiding problems and optimizing outcomes. Semin Dial. 2015;28:12–15. 25338661 10.1111/sdi.12299

[2] Chow KM, Wong KT, Szeto CC, et al. Poor flow from Tenckhoff catheter. Hong Kong J Nephrol. 2013;15(1):51–52.

[3] Peppelenbosch A, van Kuijk WHM, Bouvy ND, et al. Peritoneal dialysis catheter placement technique and complications. NDT Plus. 2008;1(Suppl 4):iv23–8. 25983982 10.1093/ndtplus/sfn120 PMC4421142

[4] Xu D, Liu T, Dong J. Urgent-start peritoneal dialysis complications: prevalence and risk factors. Am J Kidney Dis. 2017; 70(1):102–110. 28284758 10.1053/j.ajkd.2016.12.021

[5] Pethő Á, Szabó RP, Tapolyai M, et al. Bedside placement of peritoneal dialysis catheters - a single-center experience from Hungary. Ren Fail. 2019; 41(1):434–438. 31162993 10.1080/0886022X.2019.1614058 PMC6566899

[6] Yip T, Lui S, Lo W. The choice of peritoneal dialysis catheter implantation technique by nephrologists. Int J Nephrol. 2013;2013:940106. 23431443 10.1155/2013/940106 PMC3569939

[7] Maio R, Figueiredo N, Costa P. Laparoscopic placement of Tenckhoff catheters for peritoneal dialysis: a safe, effective, and reproducible procedure. Perit Dial Int. 2008;28(2):170–173. 18332453

[8] Tullavardhana T, Akranurakkul P, Ungkitphaiboon W, et al. Surgical versus percutaneous techniques for peritoneal dialysis catheter placement: a meta-analysis of the outcomes. Ann Med Surg. 2016;10:11–18. 10.1016/j.amsu.2016.07.007 PMC4961679 27489619

[9] Li PK, Szeto CC, Piraino B, et al. International Society for peritoneal dialysis. Peritoneal dialysis-related infections recommendations: 2010 update. Perit Dial Int. 2010;30(4):393–423. 20628102 10.3747/pdi.2010.00049

[10] Singh N, Davidson I, Minhajuddin A, et al. Risk factors associated with peritoneal dialysis catheter survival: a 9-year singlecenter study in 315 patients. J Vasc Access. 2010;11(4):316–322. 20890875 10.5301/jva.2010.5774 PMC3207262

[11] Moon JY, Song S, Jung KH, et al. Fluoroscopically guided peritoneal dialysis catheter placement: long-term results from a single center. Perit Dial Int. 2008;28(2):163–169. 18332452

[12] Chula DC, Campos RP, de Alcântara MT, et al. Percutaneous and surgical insertion of peritoneal catheter in patients starting in chronic dialysis therapy: a comparative study. Semin Dial. 2014;27(3):E32–7. 24118030 10.1111/sdi.12147

[13] Ladd AP, Breckler FD, Novotny NM. Impact of primary omentectomy on longevity of peritoneal dialysis catheters in children. Am J Surg. 2011;201:401–405. 21367387 10.1016/j.amjsurg.2010.08.022

[14] Kavalakkat JP, Kumar S, Aswathaman K, et al. Continuous ambulatory peritoneal dialysis catheter placement: is omentectomy necessary? Urol Ann. 2010;3:107–109. 10.4103/0974-7796.68858 PMC2955224 20981197

[15] Xie J, Kiryluk K, Ren H, et al. Coiled versus straight peritoneal dialysis catheters: a randomized controlled trial and meta-analysis. Am J Kidney Dis. 2011;58(6):946–955. 21872978 10.1053/j.ajkd.2011.06.026

[16] Hagen SM, Lafranca JA, Ijzermans JN, et al. A systematic review and meta-analysis of the influence of peritoneal dialysis catheter type on complication rate and catheter survival. Kidney Int. 2014;85:920–932. 24088961 10.1038/ki.2013.365

[17] Chow KM, Wong SSM, Ng JKC, et al. Straight versus coiled peritoneal dialysis catheters: a randomized controlled trial. Am J Kidney Dis. 2020;75(1):39–44. 31445925 10.1053/j.ajkd.2019.05.024

[18] Medani S, Shantier M, Hussein W, et al. A comparative analysis of percutaneous and open surgicaltechniques for peritoneal catheter placement. Perit Dial Int. 2012;32(6):628–635. 22550118 10.3747/pdi.2011.00187 PMC3524906

[19] Savander SJ, Geschwind JF, Lund GB, et al. Percutaneous radiologic placement of peritoneal dialysis catheters: long-term results. JVIR. 2000 11(8):965–970. 10997457 10.1016/s1051-0443(07)61323-2

[20] Liakakos T, Thomakos N, Fine PM, et al. Peritoneal adhesions: etiology, pathophysiology, and clinical significance. Recent advances in prevention and management. Dig Surg. 2001;18(4):260–273. 11528133 10.1159/000050149

[21] Stanciu D, Menzies D. The magnitude of adhesion-related problems. Colorect Dis. 2007;9(s2):35–38. 10.1111/j.1463-1318.2007.01346.x 17824968

[22] Ten Broek RP, Issa Y, Van Santbrink EJ, et al. Burden of adhesions in abdominal and pelvic surgery: systematic review and meta-analysis. BMJ. 2013;347:f5588. 24092941 10.1136/bmj.f5588 PMC3789584

[23] Keshvari A, Fazeli MS, Meysamie A, et al. The effects of previous abdominal operations and intraperitoneal adhesions on the outcome of peritoneal dialysis catheters. Perit Dial Int. 2010;30(1):41–45. 20056978 10.3747/pdi.2008.00121

[24] Kothari SN, Fundell LJ, Lambert PJ, et al. Use of transabdominal ultrasound to identify intraabdominal adhesions prior to laparoscopy:a prospective blinded study. Am J Surg. 2006;192(6):843–847. 17161105 10.1016/j.amjsurg.2006.08.055

[25] Li Z, Ding H, Liu X, et al. Ultrasound-guided percutaneous peritoneal dialysis catheter insertion using multifunctional bladder paracentesis trocar: a modified percutaneous PD catheter placement technique. Semin Dial. 2020;33(2):133–139. 32160357 10.1111/sdi.12862 PMC7187385

[26] Smart J. Pneumoperitoneum-refill needle. Lancet. 1946;2(6421):420. 20998906 10.1016/s0140-6736(46)90937-3

[27] Arif A, Jan T, Tasnim K, et al. Modification of the peritoneoscopic technique of peritoneal dialysis catheter insertion: experience of an interventional nephrology program. Semin Dial. 2004;17(2):171–173. 15043626 10.1111/j.0894-0959.2004.17221.x

